# Assessment of Ochratoxin A Exposure in Ornamental and Self-Consumption Backyard Chickens

**DOI:** 10.3390/vetsci7010018

**Published:** 2020-02-07

**Authors:** Alessandro Guerrini, Alberto Altafini, Paola Roncada

**Affiliations:** Department of Veterinary Medical Sciences, University of Bologna, Via Tolara di Sopra 50, 40064 Ozzano dell’Emilia (Bologna), Italy; alessandro.guerrini5@unibo.it (A.G.); alberto.altafini@unibo.it (A.A.)

**Keywords:** backyard chickens, bile, food safety, HPLC-FLD, ochratoxin A

## Abstract

Ochratoxin A (OTA) is a mycotoxin that may be present in various food and feed of plant and animal origin, including chicken meat. In Italy, backyard poultry farming is rather widespread. Animals are raised for meat, eggs and for ornamental purpose, and they are often fed with home-made diets not subject to official controls. The purpose of this study was to evaluate exposure of ornamental and backyard chickens to OTA using biliary ochratoxin A as a biomarker. Therefore, bile samples, in addition to kidney, liver and muscle, were collected from 102 chickens reared in 16 farms located in 6 Italian regions. High-performance liquid chromatography method and fluorimetric detection (HPLC-FLD) analysis were carried out firstly on bile from all animals, and OTA was detected in 12 chickens (concentration range 3.83–170.42 µg/L). Subsequently, the kidneys of these chickens were also analysed, and the mycotoxin was not detected. The analytical detection limits (LODs) of OTA in bile and kidney were 2.1 µg/L and 0.1 µg/kg, respectively. In conclusion, these animals were exposed to OTA but their meat can be considered safe, given that this mycotoxin, if present, concentrates highest in kidneys. Biliary ochratoxin A confirms its use as a valid biomarker to assess exposure of poultry to OTA.

## 1. Introduction

Ochratoxins are known mycotoxins, secondary toxic metabolites produced by several fungal species mainly belonging to the genera *Aspergillus* and *Penicillium*. Among ochratoxin forms (nominally referred to as A, B, C), ochratoxin A (OTA) is the most prevalent and relevant of this group, with the greatest toxic effects [1]. OTA may be present in almost all cereals of zootechnical interest, such as corn, barley, rice, sorghum, oats, soya beans [2], and also in dry foodstuffs such as coffee beans, spices, cocoa [3] and raisins [4,5]. Even drinks such as wine [6], grape juice and beer [7,8] may be contaminated. OTA has also been found in foods of animal origin, such as cheese [9], hams, salami [10,11,12], and chicken meat [13,14,15,16]. Considering the wide variety of foods where OTA and other mycotoxins have been found, it can be said that humans and animals are constantly exposed to these toxins. In food safety context, the problem of residues with potentially toxic activity has grown in importance over time, and the monitoring of "natural" contaminants such as mycotoxins, has become a routine control for the agri-food industry. The International Agency for Research on Cancer (IARC) classified OTA in 2B group (possibly carcinogenic to humans) [17], and for this reason, maximum residue limits (MRL) and guidance values have been set at European level for food or feeding stuffs. For example, in unprocessed cereals for human consumption, a limit for OTA of 5.0 µg/kg has been established [18], while in cereals and cereal products for animal feeding, the European Commission set a guidance value for OTA of 0.25 mg/kg [19]. Moreover, in compound feed for poultry and pigs, two species in which the presence of this mycotoxin in edible tissues is well documented [20,21], more restrictive guidance values for OTA (0.1 mg/kg and 0.05 mg/kg, respectively) have been set [19]. However, in Italy, since 1999 the Ministry of Health has established a guidance value for OTA of 1 µg/kg only in pork meat and derived products [22]. Toxicokinetics, toxic effects, and residues of OTA have been studied in different avian species, such as turkey, Muscovy ducks, broiler chickens [23], and Japanese quail [24]. 

Poultry species are also reared in backyard farms for the production of eggs and meat for self-consumption. This type of breeding, similar to free-range breeding method, is very widespread in Italy. Despite the non-negligible role of such sector, few data are available on the real number of these small backyard holdings, also because poultry rearing below 50 heads are not registered in the National Data Base for Livestock Registration. In 2004, the production of chicks intended for backyard poultry farm was 30 million, while a more recent survey showed that in the period 2004–2014 backyard poultry rearing only partially confirmed the quantity of 2004 [25]. In addition to the farms were chicks come from poultry hatcheries, there are ornamental poultry farms in which purebred chicks born and raised on farm are bred for participation in beauty competitions, and the birds which do not conform to the breed standard are slaughtered for consumption or offered for sale live. In backyard farms, where animals are often fed with home-made diets not subject to official controls, there is no a real knowledge of the levels of exposition to OTA, and consequently of the health risks for consumers. 

The aim of this preliminary study was to evaluate the presence of OTA in purebred chickens reared for self-consumption and/or for beauty competition in backyard farms, an important and often overlooked area of Italian poultry breeding. For this purpose, biliary ochratoxin A was used as a biomarker to assess exposure of chickens to this mycotoxin, according to the study conducted by Armorini et al. [15]. Analyses were performed using the high-performance liquid chromatography method and fluorimetric detection (HPLC-FLD).

## 2. Materials and Methods

### 2.1. Farms and Animals

A total of 102 samples of bile, kidney, liver and muscle from backyard chickens of both sexes were collected to verify the presence of OTA. Sampling was performed after home-made slaughter carried out by farmers, who allowed the collection of matrices for diagnostic use. Seventeen homogeneous farms were selected on the basis of certain criteria, such as the breeds reared, the breeding method, and how chickens were fed. The farms, all situated in open countryside, were located in six different Italian regions (four in Lombardy, one in Friuli Venezia Giulia, one in Emilia Romagna, five in Tuscany, three in Lazio, two in Campania). The animals were reared with the free-range method for the production of meat and eggs for self-consumption and for beauty competitions. The chickens were of light breed (1–2 kg) and heavy breed (>2 kg), and in particular belonged to the following breeds: Leghorn, Amrocks, Barred Plymouth Rock, Robusta Lionata, Sussex, Marans, Australorp and Indian Fighter.

### 2.2. Diets and Nutrition

For this study, farms that administered very similar diets to animals were selected. In the first period of life (0–50 days of age), the chicks were fed with commercial feeds. Subsequently, the animals were fed with home-made balanced diets prepared with cereals and by-products. The diet was generally administered ad libitum, and the birds had free access to clean water. Farmers also supplied mash twice a day, 3/4 times a week. Furthermore, the diet was supplemented with kitchen waste such as bread, fruit, vegetables, and orchards. The ingredients, their average inclusion rates, and the chemical composition of the diets are shown in Table 1 and Table 2.

### 2.3. Sampling

The slaughter was carried out “on farm” by farmers. Animals were slaughtered at 6–7 months of age, depending on the breed. Bile and kidneys are considered slaughter waste, while liver is usually consumed. Immediately after removing the liver from the carcass, bile samples were collected using a 2.5 mL sterile syringe and stored in identified Eppendorf tubes. Kidney, liver, and muscle samples were placed in 50 mL Falcon tubes identified with the same code of the corresponding bile. All samples were stored at −20 °C.

### 2.4. Solvents and Reagents

The chemicals and solvents used for OTA extraction and for HPLC analysis were analytical grade or HPLC grade. The OTA standard used to prepare standard solutions for the validation of the applied methodology was obtained from Sigma-Aldrich Co. (St. Louis, MO, USA). Immunoaffinity columns used for samples purification (OchraTest™ WB) were purchased from Vicam® (Milford, MA, USA). PBS buffer pH 7.0 used during sample clean up procedure was prepared by dissolving sodium chloride (8.0 g), sodium phosphate dibasic (1.2 g), potassium phosphate monobasic (0.2 g), and potassium chloride (0.2 g) in purified water so as to obtain 1 litre of buffer. Water, acetonitrile, isopropyl alcohol, dichloromethane, and methanol were purchased from Mallinckrodt Baker B.V. (Deventer, The Netherlands); citric acid, phosphoric acid, sodium bicarbonate, sodium chloride, sodium phosphate dibasic, potassium phosphate monobasic, and potassium chloride were purchased from Carlo Erba Reagents (Cornaredo, MI, Italy); acetic acid and ethyl acetate were obtained from Sigma-Aldrich Co. (St Louis, MO, USA).

### 2.5. Chromatographic Apparatus and Conditions

HPLC analysis was performed using a C18 Onyx Monolithic column 100 mm × 4.6 mm (Phenomenex, Torrance, CA, USA) coupled in sequence to a C18 Chromolith Performance RP-18e column 100 mm × 4.6 mm (Merck, Darmstadt, Germany) in a chromatographic system consisting of a System Gold Programmable Solvent Module 126 Pump (Beckman, San Ramon, CA, USA) equipped with an HT 800 L autosampler (HTA, Brescia, Italy) fitted with a 20 µl loop. The FLD detection was obtained by means of a 821 FP fluorescence detector (Jasco, Tokyo, Japan) set at the excitation wavelength of 340 nm and emission wavelength of 460 nm. The system was computer-controlled by a Beckman Coulter 32 Karat Software (Beckman, San Ramon, CA, USA). OTA separation was archived using isocratic elution and at room temperature, under conditions similar to those adopted by Altafini et al. [21]. The mobile phase consisted of 70% of a mixture of water, isopropyl alcohol-acetonitrile and acetic acid 1% (79:7:7:7 v/v) and 30% of acetonitrile. The flow rate was set at 1 mL/min, while the injection volume was 20 µL.

### 2.6. Bile Extraction

The procedure was based on the method described by Armorini et al. [20], slightly modified. Bile samples (200 µL), after being acidified with 200 µL of citric acid 30% water solution, were extracted with 2 mL of dichloromethane in 15 mL polypropylene conical-bottom centrifuge tubes by shaking for 30 minutes on a rotary shaker. The samples were then cooled on ice (10 minutes) and centrifuged (1860× *g* for 10 minutes). The lower organic layer was transferred into a conical tube and evaporated to dryness under vacuum (Univapo Martinsried/Munich, Germany). The dry residue was redissolved in 800 µL of methanol (OTA eventually present in bile samples results diluted four times in the final analysis solution), and 20 µL injected into HPLC.

### 2.7. Kidney Extraction and Clean Up

The procedure was carried out following the method for the extraction of OTA from tissues and organs of wild boar described by Bozzo et al. [26] with some modifications. The samples for analysis were obtained by weighing 2.5 ± 0.01 g of tissue, previously thawed, in 50 mL polypropylene conical-bottom centrifuge tubes, and by adding 1.5 mL of 1 M phosphoric acid and 5 mL of ethyl acetate. The mixture was homogenized for 3 minutes with Ultra-Turrax T25 (IKA Labortechnik, Staufen, Germany), centrifuged for 10 minutes at 1920× *g*, and cooled on ice for 10 minutes; the upper organic phase was then transferred into a 15 mL conical centrifuge tube. The pellet was subsequently reextracted in the same way and, after centrifugation, the ethyl acetate extract was transferred in the tube containing the organic phase resulting from the first extraction. The combined extract was then frozen overnight and, after thawing, it was centrifuged for 10 minutes at 1860× *g* in order to separate emulsified and suspended components, which were removed. Eight mL of the extract (equivalent to a 2 g sample) were measured into a 10 mL graduated glass centrifuge tube and reduced to 2 mL in a rotational vacuum concentrator (Univapo Martinsried/Munich, Germany). The ethyl acetate extract was then back-extracted by shaking it with 2 mL of 0.5 M sodium bicarbonate solution on a rotating shaker for 30 minutes. The mixture was frozen (−20 °C) for one hour, and centrifuged for 10 min (1860× *g*) to separate the organic and aqueous layer. Finally, after removing the upper organic layer, an aliquot of the bicarbonate extract (1.5 mL) was transferred into a glass tube, diluted with 3.5 mL of PBS buffer and loaded onto an OchraTest™ WB immunoaffinity column for purification. The column was washed with 10 mL of PBS buffer and 10 ml of water. The elution of OTA was performed with 1.5 mL of methanol, and the fraction collected was reduced to dryness by means of Univapo. The residue was redissolved in 150 µL of methanol, diluted with 150 µl of water, and after vortexing, transferred to a HPLC vial for analysis.

### 2.8. Method Validation

A stock solution of OTA (500 µg/mL) was prepared by dissolving 1 mg OTA standard (Sigma-Aldrich Co., St. Louis, MO, USA) in 2 mL ethanol, and stored at −20 °C.

Working standard solutions in methanol were set up and used to obtain reference standards in methanol-water solution (50:50 v/v) at five concentration levels (1, 2, 5, 7.5, 10 µg/L), and these were analysed by HPLC-FLD to generate reference curves in solvent. Aliquots of blank samples of bile and kidney were spiked with OTA standard solutions at appropriate concentration levels in order to obtain, in the final solutions resulting from the extraction process, the same concentrations of the reference standards in solvent (1, 2, 5, 7.5, 10 µg/L). These concentrations correspond to 4× higher concentrations in bile-spiked samples, and 5x lower concentrations in kidney-spiked samples, in consideration of the dilution and concentration factor of the respective extraction procedures applied. The final extraction solutions were analysed by HPLC-FD to generate reference curves in the matrix. Calibration curves were obtained from least squares linear regression analysis, and determination coefficient (R^2^) was calculated.

Specificity was assessed by analysing blank samples and OTA-spiked samples, and by verifying the absence of interfering substances at the retention time of OTA under the experimental conditions.

OTA recovery was established by comparing peak areas of spiked samples and peak areas of pure standard solutions at the same concentration levels.

Accuracy and precision were determined via analysis of spike samples at three different levels (three replicates per concentration level, for a total of nine determinations), and were expressed as percent error (% er) and relative standard deviation (RSD) of the replicates, respectively.

The limit of detection (LOD) was calculated on the basis of a signal-to-noise ratio of 3:1 at the OTA retention time, while the limit of quantification (LOQ) was established as the lowest concentration of the calibration curves. This study was performed according to ISO 9001 requirements.

## 3. Results

### 3.1. Performances of the Method

All the calibration curves showed a linear response over the entire observed ranges, with a determination coefficient (R^2^) always >0.999. 

**Figure 1:**
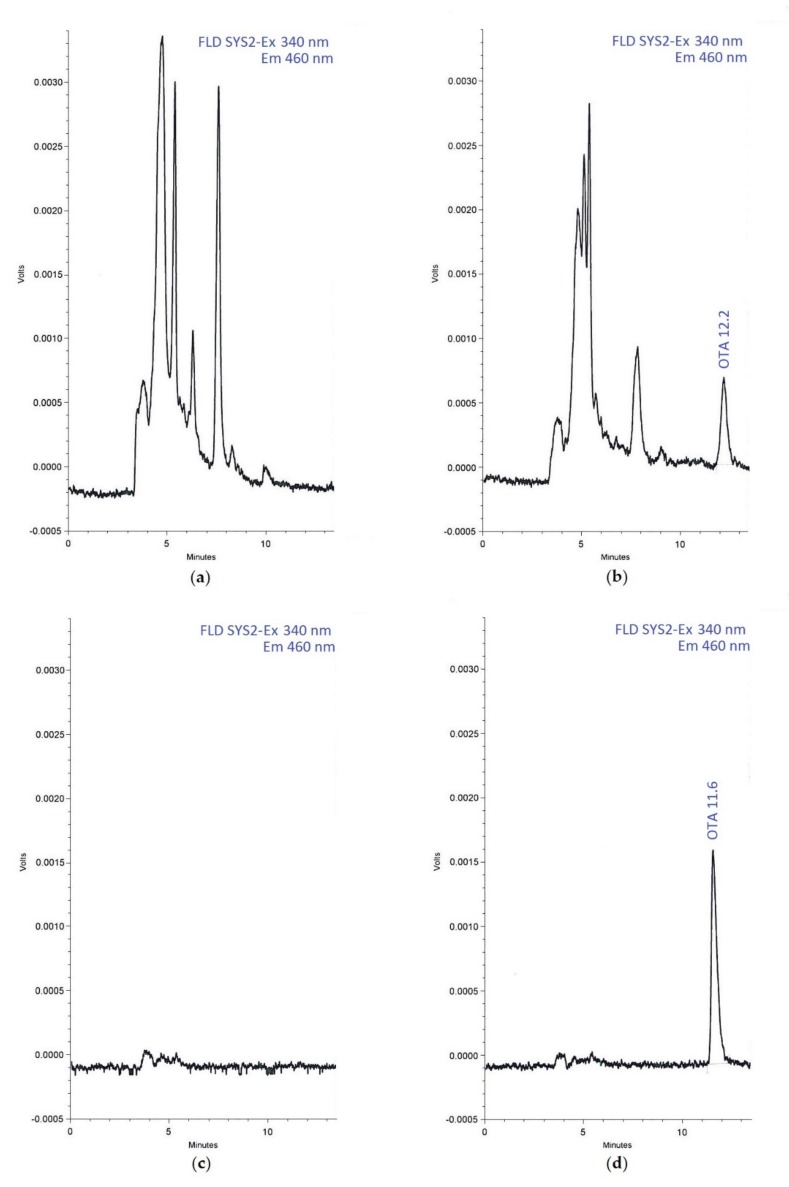
Chromatograms of a blank sample of bile (**a**), a naturally contaminated sample of bile at 13.09 µg/L level (**b**), a blank sample of kidney (**c**), a blank sample of kidney spiked with OTA at 1.5 µg/kg level (**d**).

Analysis of blank samples and spike samples of bile and kidney showed the absence of matrix interfering peaks in the retention time-window of the OTA peak. Retention time for OTA was constant both during the analysis of bile samples and kidney samples (12.2 and 11.6 min, respectively). Figure 1 shows representative chromatograms of a blank sample of bile (a), a naturally contaminated sample of bile at 13.09 µg/L level (b), a blank sample of kidney (c), a blank sample of kidney spiked with OTA at 1.5 µg/kg level (d). For the analytical session relate to bile samples, three calibration standards at concentration of 8, 20, and 30 µg/L were prepared and analysed in three different days. RSD and % er never exceeded 5.6% and 5.2%, respectively. Similarly, for the analytical session relate to kidney samples, three calibration standards at concentration of 0.4, 1, and 1.5 μg/kg were prepared and analysed in three different days. In this case, RSD and % er never exceeded 1.8% and 4.2%, respectively. 

Recovery values were estimated for each matrix at three spike levels. Three replicates were analysed for each level, and the average overall recoveries (mean of means) were 90.9% (bile) and 90.5% (kidney). The results are shown in Table 3. To determine LODs, analytical response and chromatographic noise were both measured from the chromatogram of blank samples and spike samples. The estimated LODs were 2.1 µg/L and 0.12 µg/kg for bile and kidney, respectively. The LOQ values were 4 µg/L (bile) and 0.2 µg/kg (kidney). Overall, the validation results show that the methodology applied in this study performed well in quantitating OTA in the matrices analysed.

### 3.2. Occurrence of OTA in Bile and Kidney Samples

In this study, 102 samples of bile were analysed, and 12 were positive for the presence of OTA, representing 11.8% of the total samples. OTA concentration ranged from 3.83 to 170.42 µg/L, with an average concentration of 22.25 µg/L (RSD = 211.44%). Positive bile samples and OTA concentrations are reported in Table 4. 

Excluding the farm located in Lazio (only one sample collected), the highest percentage of positive samples (38.9%, 7 out of 18 samples) was found in a farm located in Friuli Venezia Giulia (Ref. MO), and OTA concentration ranged from 3.83 to 7.29 µg/L, with an average concentration of 5.10 µg/L (RSD = 25.77%). High levels of OTA were found in bile of animals from a farm located in Tuscany (Ref. FI) in which three out of nine bile samples were positive (33%) with a concentration range between 7.54 and 170.42 µg/L (average concentration 65.26 µg/L, RSD = 139.76%). The other two positive samples (concentrations 13.09 and 22.39 µg/L) were from animals reared in two farms, one located in Lombardy (where six samples were collected), and one in Lazio (only one sample collected). OTA positive bile samples were from females only. The kidneys of the animals whose bile was found positive for OTA were analysed, but the mycotoxin was not detected in any sample tested. Kidney is generally considered the tissue of choice to assess exposure of chicken to OTA. According to the study conducted by Armorini et al. [20], extremely low concentrations of OTA in kidney (in this study, below the detection limit of 0.12 µg/kg) do not correspond to detectable levels of OTA in liver. Indeed, the tissue distribution in chicken follows the order kidney > liver > muscle > fat [27,28], as reported also for pig [27,29], rat and goat [27]. Therefore, no further investigations were carried out on the liver and muscle of those animals whose kidneys were negative for OTA.

## 4. Discussion

Analytical performance results proved that the method adopted was fit for the determination of OTA in the analysed samples, and generated reliable results. Chromatography is the most commonly used method for mycotoxin analysis in food and feed, although alternative fast, easy to use and cheap technologies have recently been developed, such as analytical methods based on biosensors [30]. However, in the case of the analysis of bile for the detection of OTA, the analytical method adopted is fast and cheap. In fact, the sample preparation procedure is a simple liquid-liquid extraction that does not need a clean-up step with SPE or immunoaffinity columns. Furthermore, due to natural OTA fluorescence, sample derivatization is not necessary for fluorescence detection. For these reasons, the adoption of the HPLC-FLD method for analysis of OTA in bile, used as biomarker of ochratoxin exposure in poultry, was an appropriate choice. 

As widely reported by the scientific literature, OTA contaminates different foods of both vegetable and animal origin, for human consumption or used as feed for farm animals. To date, there is little information and data on its presence in poultry products, and particularly in backyard chickens destined for self-consumption. In fact, these products are not subject to official controls. Furthermore, chicken products do not have any maximum residue limit for OTA. The bio-monitoring investigation conducted in these hobby farms has shown that ornamental and self-consumption poultry can be exposed to this mycotoxin. In poultry, dietary intake of OTA can lead to nephrotoxic, hepatotoxic, teratogenic and immunosuppressive effects [31]. The animals can show clinical signs such as reductions in weight gain and pigmentation, enteritis, diarrhoea, and intestinal breakage during processing from intestinal fragility [28]. Furthermore, birds’ behaviour is depressed [32], and reduced feed intake and egg production can be observed [33,34,35]. All animals considered in this study were healthy without visible adverse symptoms. No gross alterations of kidneys and liver (such as swelling, discoloration, congestion, increase in size and weight) attributable to mycotoxin ingestion were observed in chickens whose bile was found positive for OTA. Biliary ochratoxin A reconfirms its use as a valid biomarker to assess exposure of poultry to OTA, as reported by Armorini et al. [20], and as described also for the rat [36]. In kidney, which is generally considered the tissue of choice for the evaluation of OTA, the toxin was not present at levels above LOD; this is an indicator of the healthiness of the meat of these animals. However, the results of the present study also show that the presence of OTA in backyard chickens is possible, and this constitutes a potential food risk for the consumer that must not be underestimated. In factory farms, laying hens and broilers are typically reared in sheds, and the possibility of coming into contact with OTA is exclusively through feed. The tested animals were raised on pasture (meadow-orchard), with a method very similar to free-range. The fact that birds had the opportunity to wander freely, to peck and scratch in the grass at will, taking food from pastures and orchards (fresh fruit and fruit waste), and feeding also kitchen waste, suggests that these animals were potentially exposed to other possible sources of contamination by OTA, different from the feed administered daily, such as cereal grains or mash. 

With respect to OTA levels detected in bile, some further considerations should be made. Slaughtered animals had been fasting for at least 12 hours, and the gallbladders had different amounts of bile. The volume of bile might be influenced by several factors, such as the transit time through the digestive tract and the intestinal content. These factors could have affected the presence and concentration of OTA in the bile of the animals of the same slaughter group. This could explain why not all animals belonging to the same slaughtering group, consuming the same feed in the last two months of pre-slaughter life, were positive for OTA. The groups of slaughtered animals were composed of males and females of different breeds, with different food intake and slaughtering weight. The 12 OTA-positive bile samples, were from females only, six/seven months old, belonging to the following breeds: Sussex Columbia (n = 4), Barred Plymouth Rock (n = 3), Barnevelder (n = 1) and Robusta Lionata (n = 4). It can be hypothesized that the finding of the mycotoxin only in females, might be linked to hormonal factors and/or physiological factors related to eggs deposition. In fact, hens start laying eggs at 6–7 months old, depending on the breed. The deposition of eggs causes a strong metabolic effort, involving the liver in particular, with production of albumins and fat metabolization, which could affect the biotransformation of OTA. Dietrich et al. reported that some of the sex differences in distribution and elimination kinetics of OTA that have been found in various species could be due to sex differences in plasma proteins binding and/or sex variations in the expression levels of OTA-specific transporters able to modulate organ and cellular OTA concentrations, and therefore to have a direct influence on the toxicodynamics [37].

Scientific literature reports several experiments in which tissues and organs from poultry experimentally exposed to OTA were analysed [20,38,39,40,41] but only few survey studies were carried out to monitor the presence of OTA in tissues of animals normally reared on poultry farms. 

Schiavone et al., in a survey for OTA conducted on 94 chicken blood samples collected in ten poultry farms located in Italy, report 53% of positive samples with values of OTA in the range 0.003–0.165 ng/mL [42]. Milićević et al. examined 90 liver, kidney and gizzard samples coming from chicken farms located in Serbia. OTA was detected in 23 (38.33%), 17 (28.3%) and 16 (26.6%) samples, respectively, with concentration levels ranging from 0.14 to 3.9 ng/g in liver, 0.1 to 7.02 ng/g in kidneys, and 0.25 to 9.94 ng/g in gizzard [13]. Iqbal et al. report data on the presence of OTA in 115 chicken meat samples (including also liver) and 80 eggs samples, collected from central areas of Punjab, Pakistan. Forty-one percent meat and 35% eggs samples were found contaminated with the mycotoxin, and maximum level 4.70 µg/kg was detected in the liver part of chicken meat. The sampling included also 31 domestic chicken, and the meat of these animals (wings, legs and chest) did not show traces of OTA, indicating that backyard poultry is less exposed to this type of contamination [14]. In another survey conducted in Iraq on 60 samples of chicken meat and liver (30 samples each) purchased from Sulaimani markets, 26 (86.66%) and 17 (56.6%) samples of meat and liver, respectively, were found positive for OTA, with concentration levels in the range 0.149–4.106 µg/kg (meat) and 0.359–8.699 µg/kg (liver) [15]. A study carried out in Jordan on 72 poultry meat samples collected from different commercial sources showed that the 100% of thig and leg, liver, gizzard samples and 66% of breast samples were contaminated with OTA (range: 1.89 ± 0.07–7.68 ± 0.12 µg/kg), with the highest concentration levels in liver samples [16].

The results of the abovementioned studies generally show non-negligible percentages of positives, proving that exposure of chickens to this mycotoxin is quite common in many countries. Furthermore, taking as reference the Italian guideline value set for OTA in pork meat products (the only food products of animal origin for which a guideline value for OTA was established), these surveys reports OTA levels in many cases above 1 µg/kg. On the other hand, many other investigations focused on the occurrence of OTA in poultry feed and feed ingredients have shown high percentages of contaminated samples [43]. In poultry hobby farms, bad husbandry practices such as feeding chickens with moulded kitchen waste (e.g., bread) and other possible sources of moulded feed (e.g., feed from own production, generally not submitted to any compliance control) can lead to exposure of animals to OTA [44]. Our results, if compared with those from the survey studies reported above, would seem to indicate that backyard poultry is less exposed to this type of contamination, according to the findings of Iqbal et al. [14].

## 5. Conclusions

In conclusion, the present survey on the occurrence of OTA in backyard chickens showed that only a low percentage of animals was exposed to the mycotoxin and, although also high levels of OTA were found in positive bile samples, the corresponding kidney samples were negative. Consequently, it is possible to state that the consumption of meat from these animals is safe. 

On the other hand, the present research also showed that contamination by OTA could potentially occur. For this reason, hobby breeders should be appropriately sensitized with respect to the importance of the correct conservation of feeds and good breeding practices. Furthermore, health authorities could encourage the establishment of a guideline value for OTA also in chicken meat (as was done in Italy for pork meat) for a better protection of consumer health. To the best of our knowledge, the present study is the first survey carried out in Italy to assess ochratoxin A exposure in backyard poultry farms. We believe it would be interesting to continue the research extending it to other farms to have a more complete picture of the ochratoxin A risk in this type of breeding.

## Figures and Tables

**Table 1:** Feeds used in diets and inclusion rates.

Ingredients	Inclusion Rates (%)
Maize (in the form of grain, flakes or flour)	30–60
Hulless barley	12–15
Barley flaked	9–12
Wheat	25–30
Wheat bran	7–10
Wheat feed (8.5% CF)	9–10
Soyabean meal, ext. 43%–45%–46% CP	20–30
Oat flakes	5–10
Sunflower naked	3–5
Milk whey or buttermilk	1 l/kg of dry feed
Salt	0.1–0.2
Vinegar (white or apple vinegar)	10 ml/l of water used to prepare mash
Olive oil	2–5
Seeds oil (peanuts or sunflower)	2–4
Multi-vitamins and oligoelements supplement	1–1.5

**Table 2:** Chemical composition of different types of diet as formulated and fed.

Type of Diets	CP (%)	RL (%)	CF (%)	ASH (%)	LYS (%)	MET (%)	Met + Cis (%)	Ca (%)	Energy (kcal/kg)
Dry grain diet	16–17.5	3.5–4	4–7	9.5–11	0.85–1.1	0.30–0.40	0.48–0.55	0.80–1	2.900–3.100
Mash	16–18	3.5–8	4–8	9.5–12	0.85–1.1	0.30–0.40	0.50–0.55	0.80–1	2.900–3.300

CP: Crude Protein; RL: Raw Fat and Oil; CF: Crude Fibre; ASH: Ash; LYS: Lysine; MET: Methionine; Met + Cis: Methionine + Cysteine; Ca: Calcium; kcal/kg: kilocalorie/kilogram.

**Table 3:** Recovery data of the method for analysis of OTA in samples of bile and kidney spiked at three concentration levels.

Matrix	OTA Spiking Levels (µg/L or µg/kg)	Recovery (%) ^1^	M (%) ^2^
Bile	8	87.1	90.5
	20	89.3	
	30	96.3	
Kidney	0.4	90.5	90.9
	1	91.1	
	1.5	89.8	

^1^ Number of replicates: 3. ^2^ Average recoveries of the three spiking levels.

**Table 4:** Samples of bile tested positive for OTA.

Farm (ref.)	Region	Total Samples	F	M	Positive Samples (ref.)	OTA (µg/L)	Sex	Positive Samples/Total Samples	Positive Samples (%)
MO	Friuli Venezia Giulia	18	9	9	MO 12	5.36	F	7/18	38.9
					MO 13	3.83	F		
					MO 14	3.85	F		
					MO 15	7.29	F		
					MO 16	4.62	F		
					MO 17	6.36	F		
					MO 18	4.38	F		
FI	Tuscany	9	4	5	FI 19	7.54	F	3/9	33.3
					FI 20	17.83	F		
					FI 22	170.42	F		
LO	Lombardy	6	4	2	LO 6	13.09	F	1/6	16.7
RO	Lazio	1	1	0	RO 1	22.39	F	1/1	100

F, females; M, males
